# Web-Based Patient Recommender Systems for Preventive Care: Protocol for Empirical Research Propositions

**DOI:** 10.2196/43316

**Published:** 2023-03-30

**Authors:** Pamella Howell, Arun Aryal, Crystal Wu

**Affiliations:** 1 Department of Information Systems College of Business and Economics California State University, Los Angeles Los Angeles, CA United States

**Keywords:** recommender systems, preventive care, health information systems, eHealth, clinical quality measures

## Abstract

**Background:**

Preventive care helps patients identify and address medical issues early when they are easy to treat. The internet offers vast information about preventive measures, but the sheer volume of data can be overwhelming for individuals to process. To help individuals navigate this information, recommender systems filter and recommend relevant information to specific users. Despite their popularity in other fields, such as e-commerce, recommender systems have yet to be extensively studied as tools to support the implementation of prevention strategies in health care. This underexplored area presents an opportunity for recommender systems to serve as a complementary tool for medical professionals to enhance patient-centered decision-making and for patients to access health information. Thus, these systems can potentially improve the delivery of preventive care.

**Objective:**

This study proposes practical, evidence-based propositions. It aims to identify the key factors influencing patients’ use of recommender systems and outlines a study design, methods for creating a survey, and techniques for conducting an analysis.

**Methods:**

This study proposes a 6-stage approach to examine user perceptions of the factors that may influence the use of recommender systems for preventive care. First, we formulate 6 research propositions that can be developed later into hypotheses for empirical testing. Second, we will create a survey instrument by collecting items from extant literature and then verify their relevance using expert analysis. This stage will continue with content and face validity testing to ensure the robustness of the selected items. Using Qualtrics (Qualtrics), the survey can be customized and prepared for deployment on Amazon Mechanical Turk. Third, we will obtain institutional review board approval because this is a human subject study. In the fourth stage, we propose using the survey to collect data from approximately 600 participants on Amazon Mechanical Turk and then using R to analyze the research model. This platform will serve as a recruitment tool and the method of obtaining informed consent. In our fifth stage, we will perform principal component analysis, Harman Single Factor test, exploratory factor analysis, and correlational analysis; examine the reliability and convergent validity of individual items; test if multicollinearity exists; and complete a confirmatory factor analysis.

**Results:**

Data collection and analysis will begin after institutional review board approval is obtained.

**Conclusions:**

In pursuit of better health outcomes, low costs, and improved patient and provider experiences, the integration of recommender systems with health care services can extend the reach and scale of preventive care. Examining recommender systems for preventive care can be vital in achieving the quadruple aims by advancing the steps toward precision medicine and applying best practices.

**International Registered Report Identifier (IRRID):**

PRR1-10.2196/43316

## Introduction

### Current State of Recommender Systems in Health Care

The integration of recommender systems into eHealth or e-medicine is an underexplored area in research [1]. With the abundance of medical information available on the web, expertise and context-specific knowledge are needed to analyze it effectively [2]. Implementing technology such as recommender systems in health care services can bring about several benefits. First, it can enable aging population groups such as baby boomers, Generation X, and millennials to effectively process and filter the information they find on the web or receive from medical professionals [3]. Second, it can assist insurers and health care providers to shift toward a merit-based payment system, in which providers are compensated based on their patients’ outcomes and the ability to lower costs while keeping patients healthy [4]. To facilitate this transition, recommender systems will act as part of the patient-centered team-based workflow, by supporting patient compliance with the treatment protocol. This shift can encourage providers to adopt a preventive approach, rather than just treating secondary and tertiary medical conditions.

### Objectives

This study examines the extant literature and formulates quantitative propositions to explore the factors influencing patient use of recommender systems for preventive health decision-making. A proposition is a statement formulated for empirical testing that may be true or false based on some observable phenomena. It also proposes a study design and methods for developing a survey and conducting an analysis. Preventive care helps patients to identify diseases that can cause medical problems before they become serious. Many Americans are unaware of simple tests that could save their lives or prevent years of costly treatment [5,6]. For example, the US Preventive Services Task Force recommends initiating low-dose aspirin use for the primary prevention of cardiovascular disease. In 2019, the Centers for Disease Control and Prevention reported that stroke (cerebrovascular disease) was the fifth leading cause of death in the United States. Patient use of recommender systems can predict and enhance the treatment protocol for diseases such as stroke [7] and could reduce the number of deaths.

The existing literature lacks information about the factors motivating patients to use recommender systems for preventive care. The propositions developed in the study are intended to assist patients in deciding whether to use the available recommender systems for preventive care. We also aim to provide a starting point for future researchers interested in using recommender systems for preventive care. We begin this inquiry into the eHealth recommender system by describing the types of recommender systems. We develop practice-based empirical propositions and further the applied research angle by identifying salient issues with recommender systems. Finally, we discuss general issues with recommender systems and provide strategies to address them to improve the use of recommender systems.

### Recommender System Types and Applications

#### Overview

Traditional recommender systems can be classified as collaborative, content based, and hybrid [8]. Recommender systems also use the following algorithmic approaches to recommend items: group and knowledge-based recommendations [9] and demographic filtering [10]. Finally, there is a context-aware filtering classification.

#### Collaborative Filtering

Collaborative filtering is the most used type of recommender system built on a collaborative algorithm that uses 1 user’s choice to predict subsequent user preferences [11]. Correlation among users plays an essential role in this type of recommender system. Collaborative filtering applications are heavily used in e-tourism, where the preferences of previous visitors are used to predict activities that new travelers may engage in [10]. In addition to traditional collaborative filtering methods, *matrix factorization* improves the algorithm by using a baseline [12]. Other proposed enhancements to the collaborative filtering method include using trust as a replacement for the evaluation performed by a user. Results from studies indicate that using trust as a filler for rating preserves the accuracy and coverage of the predictions provided by the recommender systems [13]. To improve customer resource management, researchers modified the collaborative filtering method to include a multifactor for a bipartite network—that is, an affiliation network. The proposed model improved the recommendations’ accuracy and diversity [14].

Researchers have proposed a hybrid method to address the limitations of the traditional collaborative filtering approach. By incorporating the Big 5 personality dimensions, the collaborative filtering algorithm’s active learning capabilities are enhanced, allowing for the use of personality type to overcome the problem of sparsity, resulting in improved accuracy and a great number of recommendations [15]. Other modifications include developing new systems that can learn and adapt to user feedback to elicit changes in health behaviors. For example, researchers have proposed a collaborative filtering-based computer-tailored health communications system that delivers personalized messages to individual patients based on explicit feedback (ratings) about past messages. These studies suggest that most users have positive opinions about smoking cessation support messages recommended based on their past ratings [16]. Another example of adapting to user feedback is creating an algorithm to recommend treatment based on patients’ pretreatment preferences and posttreatment satisfaction. This study demonstrates the feasibility of using a collaborative filtering recommender system to provide accurate recommendations and guide shared decision-making [17].

Furthermore, collaborative filtering and clustering can be used to develop a drug recommendation system that suggests medications for patients with diabetes by comparing a specific patient profile with those of patients with similar characteristics [18]. Similarly, collaborative filtering and clustering can be used to develop a recommender system for patients with cardiovascular diseases [19]. Although the number of studies on collaborative filtering systems is increasing, their application in the health care domain remains limited. The use of this type of recommender system for computer-tailored interventions and health recommendations is still in its early stages. However, there is potential for these systems to determine appropriate drugs and treatments for patients and deliver personalized messages that motivate behavior change.

#### Content-Based Filtering

Content-based recommender systems (CBRSs) rely primarily on item descriptions [20]. They evaluate options and recommend a product, person, or service based on a user’s previous acquisitions or suggested preferences [21]. The process of a CBRS is divided into 3 steps. First, the content is represented in a processable format. Then, the algorithm learns about the user. Finally, a recommendation is made for that specific user. The advantage of a content-based system is that it offers personalized recommendations and does not require a rating or previous documentation of an item, thus making the recommendation process more efficient [22]. However, the complexity of the current e-service and e-product landscape requires CBRS users to integrate it with other recommender algorithms.

CBRSs have a range of applications in health care, such as promoting data set reusability, refining the research process, and recommending health education. They accomplish this by streamlining the process of identifying relevant data sets. For example, by deducing researchers’ academic interests from their list of publications, the system can make data set recommendations that users may find useful or interesting [23]. In addition, the process of conducting clinical research studies is refined by calculating the similarity between annotated publications and the research query and recommending the most relevant literature. This enhanced recommender system can be integrated into research platforms to support future medical studies by helping researchers refine their research questions [24]. Furthermore, CBRS can be used to develop a content-based system that recommends health educational websites to users based on the metadata of the videos they have watched. The system suggests relevant websites for a given health video and facilitates the search for additional related content [25]. Although existing studies propose novel approaches, they have yet to be implemented in the broader health care context, and their limitations warrant further exploration. However, as the volume of health-related information increases, solutions that assist users in selecting relevant information become increasingly important. This recommender system can support health information retrieval by reducing the burden of searching for relevant information.

#### Hybrid Filtering

The hybrid filtering recommender system aims to compensate for the shortcomings of a classification model or enhance others with the advantages of an algorithm [21]. Hybrid recommender systems are a special type that combines ≥2 recommendation strategies. For example, researchers combine collaborative and knowledge-based filtering systems to create hybrids in the restaurant industry. When using hybrid systems, rich data, such as how a particular food helped reduce weight [26], can act as a filtering mechanism for group members. Results show that semantic knowledge-based ratings improve the collaborative filtering model [27]. Subsequent studies build upon previous findings by including an additional contextual modification that improves the performance of the recommender system [27]. Traditional methods of accessing videos, audio, and television have changed; a technological development is IP television, which streams content over proprietary IP-based networks. The recommender systems proposed by researchers suggest videos to users, which they would not have selected otherwise. Results show that recommender systems provide valuable recommendations despite high demand [28]. Most models today are developed as hybrids because they are more robust.

An example of hybrid collaborative filtering is a system that recommends search terms for a given patient to clinicians. The proposed hybrid model outperforms top-N recommendations by improving the efficiency and effectiveness of retrieving relevant patient information [29]. Another example is a Drug Development Recommendation model that supports pharmaceutical companies in determining which drug groups to develop. The Drug Development Recommendation model supports decision-making before companies initiate new drug development and improves the success rate by making recommendations tailored to a specific company and its unique business conditions. Another hybrid recommender system was developed to generate peer and content recommendations for patients with diabetes based on their profile, health status, and ratings of personalized content. This development offers a glimpse into the potential of recommender systems to promote a positive, healthy lifestyle via the implementation of a personalized, mobile peer support system [30]. The applications of hybrid recommender systems in health care are emerging; however, they are not used extensively in the health domain. The number of published studies on the use of hybrid models in health care is minimal so far. However, the limitations of content-based and collaborative recommender systems suggest that new studies will likely explore applications of the hybrid approach further. 

#### Group Recommendation

Group recommendations are based on the collaboration of an entire group [9,31]. Alternatively, some refer to this recommendation type as community based [32]. An individual’s community may include colleagues, friends, and family; members typically have a common outlook. Expansions in this area include travel and location-based services, where the trust walker algorithm enhances users’ perception of the reliability of location recommendations given [32]. Another study concentrated on folk recommendations with a tripartite composition, suggesting that this type of filtering connects users with the same interests via social tagging; a well-known example of this recommender system is Flickr [33]. The group recommender system matrix is sparse, and users’ preferences are unknown. Sparsity is a problem in a group recommender system matrix, and researchers developed a memory-based technique to resolve the sparsity issue. The results indicated that the proposed method performs better than existing approaches in generating group recommendations [34].

Group recommender systems support decision-making by integrating the preferences or attributes of members. There is an increase in the number of studies that evaluate group recommender systems; however, their exploration remains low in the eHealth and e-medicine categories. Previous studies have assessed the composition of a group and ways to optimize the accuracy of recommendations. A study found that personality could improve the accuracy of predictions [35]. Another network-based recommender algorithm in an Italian health care organization computed similarities among patients. It generated a ranked list of physicians and hospitals suitable for a given patient profile using health data shared by the community [36]. It will be challenging to generate a real group consensus in the health care domain; however, studies show that there are ways for group recommender systems to achieve a high level of agreement among their members [37].

#### Context-Aware Recommender Systems

The context-aware recommendation system (CARS) category is relatively new; it modifies the content-based or collaborative filtering classes. This category is noteworthy owing to its potential for expanding research on recommender systems. Certain factors define CARSs, some of which include purchase purpose, location, and time. The e–health care, e-music, and e-travel domains successfully use the CARS algorithm. Change over time is an assessment of contextual factors that points to change over time to determine static or dynamic elements. Factors are assessed based on what is known about the facet and whether it is unobservable, partially observable, or fully observable. There are 3 forms of context-aware recommendations (prefiltering, postfiltering, and context modeling), which include the context as part of the rating process [38]. Adding context and trust enhances the collaborative filtering algorithm to combat the sparsity problem. The results from studies including these modifications indicate better recommendations [39]. A prefilling contextual technique referred to as item splitting is used to separate the characteristics of an item into a minimum of 2 distinct contextual factors; this is another method of improving the sparsity problem [40]. Hybreed is a tool developed to integrate context in the recommender system design [41]. Using accuracy and diversity as performance measures, a comparison of the 3 forms of contextual recommendation models indicates that the task affected both performance measures. Therefore, further studies in this area are needed, as no attempts have been made to reproduce the 3780 experiments conducted in this study [42].

A detailed review of context awareness and health awareness in eHealth revealed that both factors are essential for developing user-personalized recommender systems [43]. Considerable amounts of data have accumulated in clinical databases. Recommender systems can supply patients with basic descriptions of information in health records to facilitate the comprehension of their health status. A study hypothesized whether the retrieval precision of an advanced health recommender system was higher than that of a naive system. The results indicated that advanced health recommender systems enhanced patient understanding of health records more than a naive system [44]. Researchers from a separate study proposed a cloud-based hybrid predictive model for a personalized health information service. Experiment results discovered that participants have a positive response toward usability measurement dimensions [45-47] of the system, such as perceived usefulness, expectation confirmation, perceived value, satisfaction, and perceived trust [48].

## Methods

### Overview and Workflow

**Figure 1:**
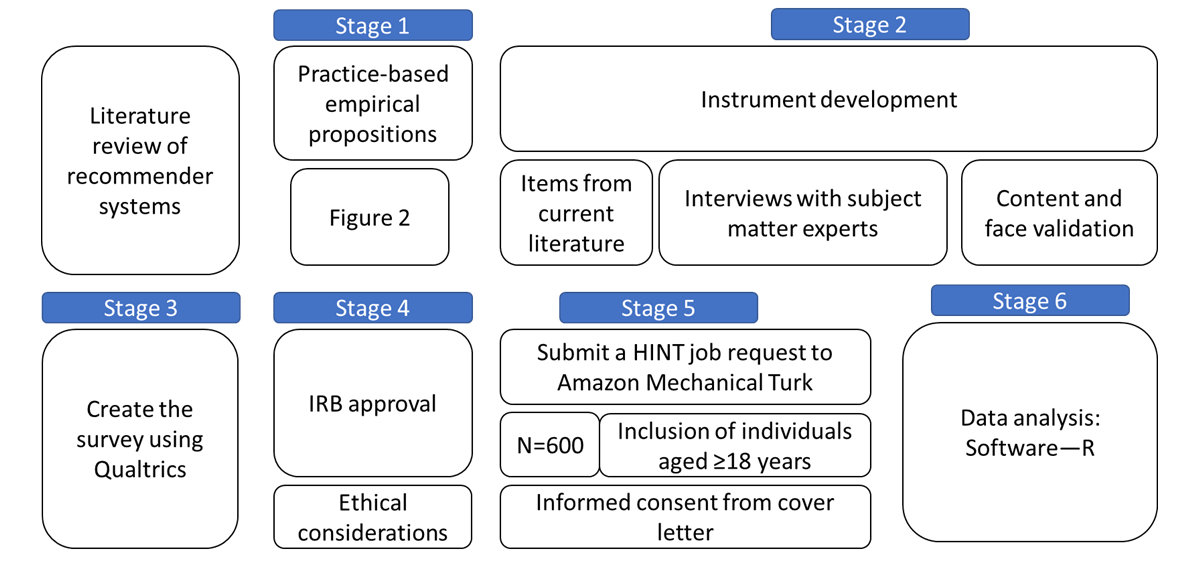
Study design and workflow. IRB: institutional review board; HINT: Human Intelligence Tasks.

This study will use a multistage qualitative methodology to explore patient perceptions about using recommender systems for preventive care decisions. Stage 1 of the framework focuses on developing practice-based empirical propositions. Stage 2 includes the development of survey instrument. Then, we will obtain institutional review board (IRB) approval for the study in stage 3, before data collection and analysis can be conducted in stages 4 and 5, respectively (Figure 1).

### Stage 1—Practice-Based Empirical Propositions

#### Overview

Although recommender systems are seen as valuable in health care, no studies synthesize the factors leading patients to use this technology. The following sections present the propositions developed from constructs in previous studies that may influence patient recommender systems. Examining factors influencing the use of this technology is vital because it will support achieving the quadruple health aims—improving the patient population, reducing the cost of care, enhancing the patient experience, and improving provider satisfaction [49].

#### Privacy

Privacy is an issue within the literature on recommender systems [50]. Privacy concerns stem from the need for users to disseminate personalized information to allow for the best recommendation [51]. Researchers have developed methods to combat privacy challenges; however, this factor may still affect the patient’s perception of usability. Strategies to protect privacy include the secure processing architecture and the anonymous contributions architecture [52]. Another attempt to reduce privacy concerns in recommender systems is justification messages to improve the experience with a recommender system [53]. A recommender system can also assist individuals in making decisions regarding their privacy by providing them recommendations and warnings based on their privacy preferences [54].

Privacy concerns present unique challenges when adopting technology in health care. For example, users of wearable medical devices are vulnerable to data security and privacy protection issues [55]. Wearable technology data security can be classified into technical security, data management, or laws and regulations. Privacy concerns challenge mental health care providers with each advancement in technology used to deliver psychotherapy services [56]. Although using email, SMS text messaging, telehealth, electronic medical records, and mobile apps aid mental health providers, such technologies simultaneously increase the risks of unintended breaches of confidentiality. As privacy and confidentiality are regarded as essential considerations in mental health care, telepsychology is limited by concerns around privacy. To address these concerns, several researchers develop a risk assessment tool to assess whether health ITs (HITs) comply with privacy policy standards [57]. They find that users of HITs emphasize the need for transparency and policies that define the type of data collected and used. Thus, as privacy risks emerge as a common concern for health care professionals and patients, privacy issues undermine users’ confidence and willingness to use HITs. Privacy concerns affect the adoption of technology in health care. Therefore, maintaining the security and privacy of protected health information will be crucial for the future of health care.

*Proposition 1*—Privacy concerns may negatively affect the use of health care recommender systems for preventive care.

#### Trust

A social network of interlinked nodes connected by their confidence relations is a trust network [58,59]. Trust-based models help resolve sparsity problems [60]. Modifying existing recommender system classifications with trust improves ratings’ accuracy [61]. An early study on peer production services revealed that trust-based recommender systems can substantially enhance services and overcome the issue of information overload [62]. Trust can be aggregated into ontology and used to update user profiles to improve the recommendation process [63]. In scenarios where patients or individuals face conflicting recommendations, trust can help the user to make a better choice regarding the accuracy of the results [64]. Trust represents a vital antecedent of the acceptance and use of technology in health care. A recent study identifies perceived usefulness, usability, training, and technical support as trust facilitators. In contrast, the lack of privacy, perceived risks, and cost and security issues represent the main barriers [49]. Similarly, another study analyzes the enablers and impediments of trust in digital health. These trust factors indicate whether to place trust in digital health technologies [65]. Asan et al [66] focus on clinicians as primary users of artificial intelligence (AI) systems and how trust shapes their use and adoption of AI. In this context, trust refers to applying AI-based systems to support clinical decision-making. The lack of trust in AI deters users’ adoption of this technology in health care. As trust is the most notable factor influencing the adoption of wearable health devices [67], understanding how users define trust in the health care technology context provides insight into the use of technologies.

*Proposition 2*—Trust may positively affect the use of health care recommender systems for preventive care.

#### Effectiveness

An effective recommendation will help a customer make a better choice or choose a product that they would otherwise not have selected. Studies have shown that a combination of rating and content can increase effectiveness [68]. Systems can provide less effective measures by overestimating or underestimating a recommendation for a particular patient [69]. Variations in predictions should be carefully considered during the development of a health system. For preventive care measures, overestimation may cause the most ripple effects. For example, a test ordered very frequently could increase costs, or inaccurate preventive measures could have considerable medical consequences. The lack of effectiveness is an issue when adopting technology in health care. For example, technological tools, including telehealth, mobile app, and SMS text messaging, can address the mental health crisis during the pandemic. Each tool helps provide the needed psychological support; for instance, the effectiveness of telehealth is comparable with that of in-person counseling services [70]. A similar study assesses the effectiveness of mobile health (mHealth) technology in suicide prevention. The results suggest that these mHealth technology tools effectively reduce suicide-specific outcomes [71]. In addition, digital health technology effectively monitors and treats asthma [72]. Ineffectiveness is a considerable adoption barrier because it indicates the future utility of a given technology in health care.

*Proposition 3*—Effective recommendations positively affect the use of health care recommender systems for preventive care.

#### Accuracy

Accuracy is one of the most frequently used measures for evaluating a recommender system; however, these often include complex calculations that average customers are not privileged to access. The patient’s perception of whether the system is making correct predictions may influence use [69]. Service providers use accuracy measures to help customers make better product and service selections. Will the patient’s perception that the recommendations are correct affect their opinion and usability of preventive health recommendations? Accuracy is also an issue when adopting technology in health care because the low accuracy of these technologies raises concerns regarding the threat posed to patient safety [73]. The adoption of these technologies should be carefully considered, especially if their performance is inferior to that of current practices. A recent study examines the quality of information provided by web-based symptom checkers, where physicians outperform symptom checkers regarding the accuracy of diagnostic data [73]. This accuracy concern highlights the need for further studies on symptom checkers. Users cannot solely depend on the technology; for example, the Apple Watch overestimates the value for energy expenditure of patients with cardiovascular disease but accurately measures their heart rate [74]. These accuracy issues imply that it is premature to use the Apple Watch for cardiac rehabilitation. Therefore, caution should be exercised when adopting technology with accuracy issues in health care.

*Proposition 4*—Perceived accuracy positively affects the use of health care recommender systems for preventive care.

#### User Satisfaction

User satisfaction refers to the perception of the system services after personally experiencing the system [75]. The level of satisfaction that patients receive from using community-based health care recommender systems may vary across individuals. If customers are more satisfied, they tend to implement the recommendations; the opposite is true if they are dissatisfied [48]. HIT literature reports high user satisfaction leading to increased adoption of technology in health care in various health care settings. For example, patients’ acceptance of the health technology program increased with high patient satisfaction with the digital tools incorporated into the program [76]. Patient satisfaction with telemedicine determines its long-term viability beyond the pandemic [77]. Patients experienced higher satisfaction with web-based visits than with in-person visits, which is not surprising considering that health care professionals can still address patients’ questions and concerns without compromising quality. High user satisfaction leads to great acceptability for lifestyle apps in mHealth [78]. User satisfaction indicates that a specific technology sufficiently satisfies patients; however, low satisfaction rates suggest that the barrier to adopting that technology in health care is important.

*Proposition 5*—User satisfaction positively affects the use of health care recommender systems for preventive care.

#### Efficiency

A fundamental business system requirement is efficiency. For example, one of the largest recommender systems, Google, searches in seconds. Similarly, more specialized recommender systems are expected to perform tasks quickly or reduce the time it takes to make choices [79]. Efficiency is the user’s perception that the recommendation improved decision-making [47]. When adopting HIT such as recommender systems, efficiency is important. HIT refers to technology that assists in preventing, diagnosing, and treating diseases. HITs, such as electronic health records and health information exchanges (HIEs), facilitate care coordination by making information available and providing real-time decision support to physicians [80]. HIEs enable the sharing of patient health information electronically among providers, patients, and payers across different health care settings [81,82]. Researchers have assessed the impact of HIT’s efficiency. The findings demonstrated that HIT improves efficiency by decreasing use rates [83]. A similar study found that adopting HIT enhances hospital efficiency. However, adopting HIT and increasing the employment of physicians nullifies the positive impact on hospital efficiency [84]. Finally, another study aimed to determine whether participating in HIE networks improves hospital efficiency. The author concludes that participating in HIEs improves hospital efficiency but calls for more research [85]. Similar to the implementation of other large-scale HIT, recommender systems may affect patient efficiency.

*Proposition 6*—Perceived efficiency positively affects the use of health care recommender systems for preventive care.

### Stages 2 and 3—Instrument Development and Operationalization of Variables

**Figure 2:**
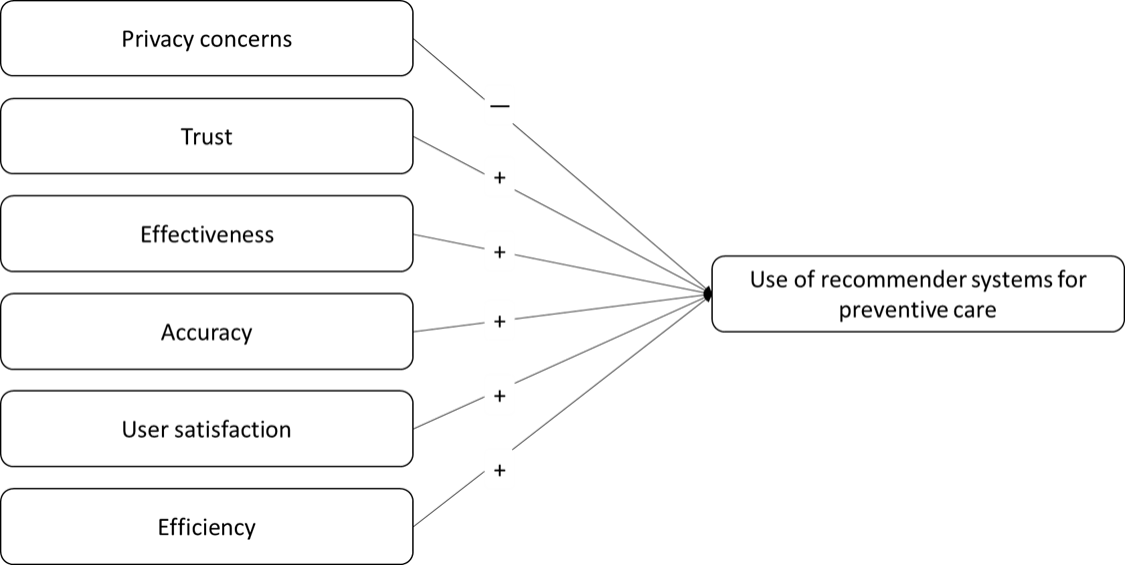
Research model—propositions for evaluating a recommender system for preventive care.

Researchers can develop an instrument after literature reviews and interviews with subject matter experts, including information systems professionals, physicians, and patients. An expert in survey design will be consulted to ensure that the questions are phrased appropriately to elicit proper responses for the measured factors. The review team can consist of 3 to 5 individuals to represent the abovementioned expertise, including the principal investigator. Before the final items are selected for the survey, we will assess content and face validity using a short questionnaire that includes the items and construct definitions [86,87]. IRB approval may not be required for this process; 25 to 30 professionals or students can be used to elicit the validation results. Items will be rated on a 7-point scale from 1 (not at all) to 7 (completely), aligned with the construct’s definition. Items that do not match precisely with the construct definitions—responses below the mean—should be refined or removed from the final instrument. Discarded questions can be replaced with new ones, and the validation process can be repeated. Figure 2 depicts the plausible measurement model for the likelihood of using recommender systems for preventive care with several reflective indicators. Table 1 presents the constructs and references.

**Table 1:** Recommender system constructs and references.

Constructs and items			Studies
**Privacy concerns **			Xu et al [88,89]
	I am concerned that the recommender system will collect large amount of personal information from me.	—^a^	
	I am concerned that the recommender system will use my personal information for unauthorized purposes.	—	
	I am concerned that unauthorized people will have access to my personal information.	—	
**Trust **			Liu and Tao [90] and Kim et al [91]
	The recommender system is trustworthy.	—	
	The content of the recommender system is reliable.	—	
	Overall, I trust the recommender system.	—	
**Competency-related trust **			Esmaeilzadeh [92], Mpinganjira [93], and McKnight et al [94]
	The recommender system is competent.	—	
	The recommender system performs its role very well.	—	
**Effectiveness **			Liu and Tao [90]
	The recommender system provides personalized recommendations that are based on my information.	—	
	The recommender system personalizes my health care experience by acquiring my personal preferences.	—	
	The recommender system delivers personalized health care services.	—	
**Accuracy **			Yang et al [95]
	The recommender system offers consistent results over time.	—	
	I feel confident that the recommender system offers error-free results.	—	
**User satisfaction **			Park et al [96]
	Overall, I am satisfied with the recommender system.	—	
	The recommender system meets my expectations.	—	
	I recommend the recommender system to others in health care.	—	
**Efficiency **			Garg et al [97]
	The recommender system will help in providing health care services.	—	
	The recommender system will increase the speed of services.	—	
	The recommender system will increase efficiency.	—	

^a^Not applicable.

The dependent variable—the use of recommender systems—can be modeled based on the theory of planned behavior using a binary item [98-100]. This theory contends that behavioral, normative, and control beliefs affect a person’s intentions to perform a particular behavior; the strength of these intentions influences an individual’s actual behavior [101,102]. This proposed study design adopts the theory of planned behavior as a fundamental behavior theory to represent patient behavior, especially in terms of their use of recommender systems. Researchers can examine patients’ adoption of recommender systems by extending the theory of planned behavior to the health care context. Individual attitudes, subjective norms, and perceived control influence patients’ intention to use recommender systems. Therefore, individual perception and social influences are antecedents of their intention to use recommender systems; the greater this intention, the more likely that the adoption behavior will be realized. The use variable can be dichotomous (yes or no).

Privacy concerns can include 3 items [88,89]. When formulating trust items, researchers can combine two trust constructs: (1) general trust [90,91] and (2) competency-related trust [92-94]. The effectiveness construct can use 3 reflective items [90]. The accuracy construct can be measured using 2 items [95]. Short-form measures have limitations; however, they are as effective at capturing a construct as extended scales when adequately designed [103]. Possible robustness checks are discussed in the *Stage 6—Data Analysis* section. Overall, 3 reflective items can be used to measure user satisfaction [96] and efficiency [97]. Scale scores can be computed as the averages of individual items and anchored to strongly agree, agree, somewhat agree, strongly disagree, disagree, and somewhat disagree. 

### Stage 4—Ethics Approval

This study has been submitted for ethics approval to the California State University Institutional Review Board (reference number: 2038900-1) through a secure web portal. Approval may take 4 to 6 weeks, depending on whether revisions are required and the frequency with which the board meets. It will be approved if the board finds that this study involves minimal risk to the participants. Participants are informed that they can skip questions they do not feel comfortable answering or choose not to participate. The survey is anonymous, and participants cannot be identified.

### Stage 5—Initial Data Collection: Amazon Mechanical Turk

#### Overview

This study will be conducted via the web; this aligns with the study’s aims to examine the perceptions of adults in need of preventive care. Data will be collected using Qualtrics (Qualtrics) via an anonymous survey link. Qualtrics is a web-based data collection tool; its functionality allows users to create a customized survey.

**Figure 3:**
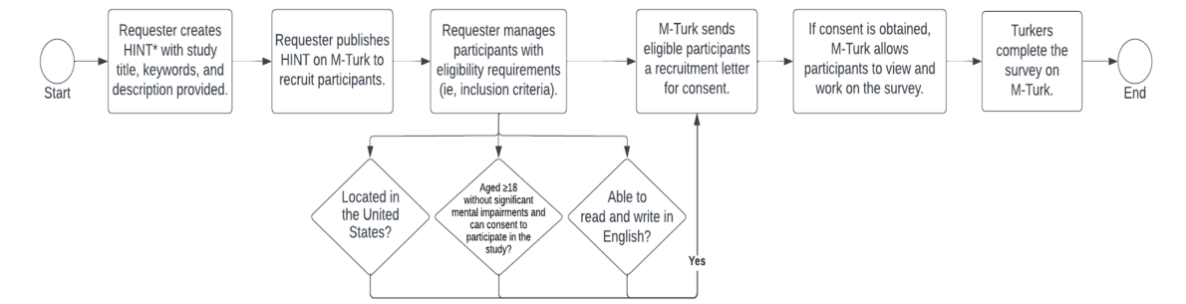
Amazon Mechanical Turk (M-Turk) workflow. HINT: Human Intelligence Tasks.

Amazon Mechanical Turk is a hosted platform that allows researchers access to a wide range of participants [104]. Participants are called *turkers* or *crowdworkers*. They browse their profile for Human Intelligence Tasks (HINT), which includes a brief description of the responsibilities. Posting the survey on the HINT list will initiate participant recruitment for the study. Amazon Mechanical Turk allows requesters to select worker requirements to enforce the inclusion and exclusion criteria. To be included, participants should be aged ≥18 years without serious mental impairments and consent to participate in the study. Participants must be in the United States and be able to read and write in English. The procedure for recruiting individuals and how participants access and complete the survey is depicted in Figure 3.

The survey cover page will be used to distribute the recruitment letter and obtain informed consent. The letter will also provide an overview of the study, participation criteria, and any benefits or risks to the participant. Completed survey data are automatically stored if Qualtrics is used. It is reasonable to assume that the time needed to complete the survey will be approximately 10 to 15 minutes. Before beginning the survey, all participants will receive a vignette [105] describing what a recommender system is and an example of how it will be used in their care.

#### Sample Vignette

Recommender systems are the systems that are designed to suggest things to the user based on many different factors. They are used by companies such as Google, Instagram, Spotify, Amazon, Reddit, and Netflix, to entice users with relevant content. A health recommender system may propose preventive measures to patients based on their clinical history and demographic characteristics. For example, based on available data, it may recommend talking to a primary care provider about the decision to initiate low-dose aspirin use for the primary prevention of cardiovascular disease.

Participants will answer 19 to 20 reflective questions to measure each construct, as outlined in Table 1. They will answer demographic questions on ethnicity, race, sex, income, education, employment, and the number of chronic conditions. No compensation will be provided for participating in the study. A sample size of approximately 600 participants may result in a medium effect size where the *r* value varies from 0.3 to 0.4. Effect size is a quantitative measure of the magnitude of the experimental effect [106].

### Stage 6—Data Analysis

Researchers can use R to analyze the research model; it is stage 5 of the proposed study design. The following robustness check can be used to evaluate the measurement instrument. Principal component analysis will be performed; only factors with eigenvalues >1 will be selected; and then, a visual assessment will be conducted using a scree plot. A parallel analysis will be completed to review the eigenvalues for the 6 factors in the conceptual model. A Harman Single Factor test can be performed to test the effect of common bias to ensure that a single factor does not emerge. To assess discriminant validity, exploratory factor analysis will be performed. Then, any factor loading <0.70 will be examined; a loading value of 0.55 is considered as good and 0.45 is considered as fair [107]. Reliability and validity will be examined using Cronbach α; a value >0.7 is satisfactory. Multicollinearity should be examined as part of the analysis; correlation should fall below the conventional threshold value of 0.6 [108,109].

To establish the reliability and convergent validity of individual items, the average variance extracted for each construct will be calculated. Composite reliability measures the overall reliability of a collection of heterogeneous but similar items. Composite reliability >0.70 threshold and extracted variance >0.50 threshold are recommended [110]. The analysis of *variance inflation factors* will determine whether multicollinearity exists among the tested variables. Variance inflation factors are a scaled version of the multiple correlation coefficients between 1 variable and the remaining independent variables. Variance inflation factors below the cutoff value of 10 indicate that multicollinearity among the variables is not supported [111]. Researchers should perform confirmatory factor analysis. The goodness-of-fit indices [112-114], adjusted goodness of fit, comparative fit index, Normed Fit Index by Bentler and Bonett [115], root mean square error approximation, and root mean square residual will be examined.

## Results

Data collection and analysis will begin after institutional review board approval is obtained.

## Discussion

This study proposes practice-based empirical propositions for developing recommender systems in health care, focusing on identifying the factors influencing patients’ use of these systems. We also provide a study design and methods for creating a survey and conducting an analysis. Our study highlights the potential of recommender systems to improve population health by educating patients about disease prevention and motivating them to use preventive services. This study aims to guide future research in the health care domain by exploring the application of recommender systems in preventive care.

Clinical quality measures such as preventive metrics are essential for monitoring health care quality. Often, patients are unaware of the many preventive quality metrics that can help improve their health care outcomes. Improved patient awareness of preventive measures helps not only them but also physicians and insurers to meet the metrics of clinical quality initiatives from advanced payment models such as primary care first, accountable care organization, merit-based incentive payment system, and Healthcare Effectiveness Data and Information Set. Hybrid recommender systems can help patients filter and use data about preventive quality metrics, making the information more accessible. Using the propositions mentioned in this paper, health care professionals and researchers can examine the factors that will lead patients to use recommender systems for preventive care.

There are advantages to implementing recommender systems and general issues that may influence patient use of the systems. As discussed in the propositions, patient privacy concerns may affect patients’ likelihood of using recommender systems. Issues stemming from privacy concerns may lead patients to not share their medical records, resulting in the cold start problem. The cold start problem affects recommender systems because new users do not have a profile, thereby limiting the ability of the recommendation systems to provide meaningful information. Another privacy concern is the intrusive nature of recommender systems [69]. Algorithms require direct feedback that is linkable to a particular user; this may engender privacy concerns. Patients may be less likely to seek recommendations if their searches or history can be linked to them by administrators.

To improve patient use of recommender systems, developers must be able to improve the trust in the systems they use or the administrators governing the data collection, processing, and dissemination processes. An issue with recommender systems is overspecification, which could result in duplicate preventive testing. Patients who receive duplicated recommendations may reduce their trust in the system’s capabilities. Duplicate testing may result in 2 issues: patient fatigue may reduce trust or overuse may increase costs. To reduce the influence of overspecification on preventive recommendations, developers may consider integrating current structured data from the patient’s medical records to train and test recommender systems.

Perceptions about the effectiveness and accuracy of recommender systems also reduce the likelihood of patients using them. A factor that could cause efficiency or accuracy issues with algorithms is the sparsity problem, in which some services have many ratings and others do not. Data accumulation is a method to rectify the sparsity problem to facilitate accurate predictions [8]. Efforts to address this data sparsity issue should include developers working closely with insurers, providers, and patients to limit the factors affecting their likelihood of sharing data. Data blocking on the physician’s side may also lead to sparsity; in response, the Cures Act should limit this practice by 2023. Furthermore, a patient may choose to share the data; however, it may not be exportable or interoperable with data from the internet or other sources. Unfortunately, data from multiple inoperable sources can lead to low efficiency. Health care organization professionals can tackle this challenge by implementing systematic data standards such as Systematized Nomenclature of Medicine, Logical Observation Identifiers Names and Codes, Current Procedural Terminology, and International Classification of Diseases and the fast health care interoperable resource standards.

This study does not address all the psychological aspects of recommender systems. Further studies are needed to better understand the physiological effects of these systems on users [69]. The trust construct developed in this study does not relate to the patient-physician trust. However, when addressing concerns about privacy and sensitive patient data, it is crucial to consider the patient-physician relationship in the design of web-based recommender systems. Developers should include features for effective communication and collaboration between patients and qualified health professionals.

Providing access to IT support services and mobile options for accessing preventive recommendations can improve patient satisfaction. Efficiency and scalability also play key roles in determining the success of recommender systems. As the amount of data available on the web continues to grow, algorithms must be able to handle the increased computational demands. Studies have shown that accuracy does not necessarily increase with large amounts of data, but computation time may [116]. Health care professionals may consider partnering with large IT companies that offer cloud computing resources to address scalability issues. Federal insurers such as Medicaid may also consider using a portion of cost savings from tracking preventive care to fund cloud computing hubs.

Additional studies are necessary on the physiological aspects of recommender systems [69]. In doing so, developers may improve user satisfaction and, therefore, the likelihood of use. Health care records can contain some of the most sensitive data about a person’s life. Addressing the intrusive nature of recommender systems and the sensitivity of patient data leveraging the patient-physician relationship is paramount. Developers of web-based recommender systems should include features for effective communication and collaboration between patients and qualified health professionals. It may not be feasible to grant patients access to their private physicians; however, allowing access to a professional may improve patient satisfaction. Recommender systems should provide, at minimum, some IT support services and offer a mobile way to access preventive recommendations [117]. Many large IT companies such as Amazon, Microsoft, and IBM have entered the health care arena; it may be opportunistic for health care professionals to partner with them to access cloud computing resources.

## Abbreviations

AI: artificial intelligence
CARS: context-aware recommendation system
CBRS: content-based recommender system
HIE: health information exchange
HINT: Human Intelligence Tasks
HIT: health IT
IRB: institutional review board
mHealth: mobile health

## Data Availability

The data sets generated during and analyzed in this study will be available from the corresponding author upon reasonable request after completion of the study.
